# The association between stress attributed to information systems and the experience of workplace aggression: a cross-sectional survey study among Finnish physicians

**DOI:** 10.1186/s12913-022-08116-w

**Published:** 2022-05-31

**Authors:** Heidi Urnberg, Kia Gluschkoff, Petra Saukkonen, Marko Elovainio, Jukka Vänskä, Tarja Heponiemi

**Affiliations:** 1 Department of Psychology and Logopedics, Faculty of Medicine, University of Helsinki, Helsinki, Finland; 2 Finnish Institute for Health and Welfare, Helsinki, Finland; 3 The Finnish Medical Association, Helsinki, Finland

**Keywords:** Aggression, Violence, Information systems, SAIS, Job demand, Work stress, Physicians

## Abstract

**Background:**

Physicians commonly suffer from workplace aggression and its negative consequences. Previous studies have shown that stressors such as job demands increase the risk of inappropriate treatment at workplace. Poorly functioning, and constantly changing information systems form a major work stressor for physicians. The current study examined the association between physicians’ stress attributed to information systems (SAIS) and their experiences of workplace aggression. Workplace aggression covered physical and non-physical aggression, perpetrated by coworkers, patients, patient’s relatives, or supervisors.

**Methods:**

A cross-sectional survey study was conducted. The participants included 2786 physicians (67.4% women) who were sampled randomly from the registry of Finnish Medical Association, which covers almost all of the Finnish physician population. First, bivariate associations were studied among participant characteristics, SAIS and workplace aggression. Logistic regression analysis was then used to further determine how SAIS was associated with the likelihood of experiencing different types of aggression.

**Results:**

Higher levels of SAIS were associated with higher likelihood of aggression with regard to all types of aggression, except non-physical aggression perpetrated by patients or relatives. The demographic factors (work-sector, gender, age) did not have a noticeable influence on the association between SAIS and aggression.

**Conclusions:**

The present results build on previous evidence on the prevalence of SAIS and its negative effects on healthcare workers. Since SAIS may increase the risk of experiencing aggression, it is possible that SAIS also endangers the wellbeing of physicians and thereby the quality of patient care. Resourcing time and training during introduction of a new IS could alleviate time pressure and thus stress attributed to managing new information systems. The role of organizational climate and general workload in arousing SAIS and aggression should be examined in future studies.

**Supplementary Information:**

The online version contains supplementary material available at 10.1186/s12913-022-08116-w.

## Background

Workplace aggression is a common and concerning occurrence within healthcare [1–4]. Healthcare professionals, including physicians, have been found to have a heightened risk of experiencing workplace aggression compared to other service workers [5], with estimated one tenth to two thirds of healthcare workers having encountered aggression [2, 5–7]. It is generally agreed that the nursing staff is at highest risk of experiencing aggression [5], but the problem is prevalent among physicians as well [2, 4]. The prevalence of workplace aggression is likely even higher than reported because the incidents tend to be underreported [2, 4], which presents an alarming picture of the state of experiencing workplace aggression in healthcare. Because the prevalence rates of workplace aggression depend on its conceptualization, it is challenging to compare prevalence estimates for aggression across different studies.

Workplace aggression can be physical or non-physical in nature and it can manifest for example as verbal abuse, physical assaults, harassment, bullying, intimidation, threatening, and obscene behaviors [6]. Consequently, there is an abundance of proposed constructs of workplace aggression seeking to define the phenomenon. However, many of these constructs lack in definition, as they show considerable overlap and do not have uniform attributes 8. Therefore, the definition of “workplace aggression” in this study will encompass simply all these manifestations and behaviors towards employees that can result in psychological, social or physical harm to the victim, as suggested in previous literature [8]. In healthcare environments, violence arises typically from patient interactions, but much of the non-physical violence experienced in the healthcare workplace is perpetrated by colleagues and superiors as well [2, 5–7]. However, studies examining workplace aggression have focused mostly on the aggression carried out by patients.

Workplace aggression has consequences for both the well-being of healthcare professionals and quality of patient care. Experiencing workplace aggression is associated with negative psychological outcomes for the healthcare worker (e.g. burnout, anxiety, depression), regardless of whether aggression is physical or non-physical [1, 3, 7]. Instances of workplace aggression have also been associated with worse quality of patient care [1, 5]. There is also evidence that physicians may deliver worse care if they are afraid of patients [5], and being a victim of aggression can change the way a healthcare worker feels and behaves around a patient [1]. Finally, workplace aggression may also have organizational consequences, as workplace aggression is associated with absences from work [1, 4] and the negative psychological consequences resulting from encountering aggression can cause some physicians to leave the organization altogether [9].

Given that workplace aggression has severe consequences, it is important to understand factors that may increase its risk. Work-related stress has been recognized as one of the factors that might increase the risk of suffering from workplace aggression from both patients and other healthcare staff [5–7]. This is especially concerning considering that work stress among healthcare workers is becoming increasingly common [10]. Indeed, rapid digitalization of healthcare has brought forth one major stressful work characteristic for healthcare workers, namely constantly changing, difficult, and poorly functioning information systems [11–14]. Information systems (IS) refer to technological systems that manage healthcare data, e.g. electronic health records, which can be used for storing, sharing, searching and retrieving digital patient information [15]. In theory, information systems can offer improvements in quality of care and access to patient information remotely [15], but it is still debated whether information systems actually hinder physicians’ job more than they help it [15, 16].

According to previous studies, information systems respond poorly to the needs of physicians, thus accumulating masses of criticism and contributing to poor well-being [11, 14, 17, 18]. Physicians’ complaints regarding information systems often include aspects related to poor functionality and usability [11, 17, 19, 20], and several IS-related factors have been directly associated with distress, stress, and burnout for healthcare professionals [13, 20]. Moreover, coping with poorly functioning or difficult to use information systems is especially difficult if there are other stressful work factors, such as time pressure [12, 19]. Unfortunately, frequently changing systems require physicians to continuously update their knowledge on information systems, and learning to use these systems requires time and training [17, 21].

There are several mechanisms as to how stress attributed to information systems (SAIS) and work aggression might be connected. First, stress in general has a negative impact on social relationships, which might lead to interpersonal conflict and aggression [22]. Emotional exhaustion, a common consequence of stress [23], can lead to depersonalization, and subsequently, to negative behavioral changes toward patients and colleagues [24]. Secondly, changing, difficult, and poorly functioning information systems may constitute an especially frustrative and stressful work-related stressor for physicians because it may hinder their work, create additional time-pressure, and interfere with patient interaction. Thus, SAIS is likely to arouse frustration and negative affect, which may create aggressive inclinations and aggressive behavior (frustration-aggression theory) [25]. Thirdly, according to social learning theories [26], people acquire aggressive behaviors by observing others. Hence, the aggressive behavior of a stressed health care worker might get modeled by patients, relatives, or other healthcare workers. Moreover, the negative affect of a physician (e.g. anxiety, irritability, anger) might influence the mood of patients, relatives, and staff (emotional contagion) [27]. This means that the negative affect of a physician might arouse negative affect in others. Indeed, it has been found that the mood of a healthcare worker is associated with the mood of patients [28] and other healthcare workers [29, 30].

Because workplace aggression is a major concern both for the wellbeing of physicians and quality of patient care, it is critical to understand risk factors that are associated with its occurrence. Previous research has shown that stressful work characteristics increase the risk of workplace aggression, potentially by inducing frustration and negative affect. Information systems have been consistently recognized as a major stressful work characteristic for physicians. However, previous studies have not, to our knowledge, examined the association between SAIS and workplace aggression. Therefore, the current study examined the association between physicians’ SAIS and their experiences of workplace aggression (physical and non-physical) perpetrated both by patients and healthcare staff.

This study aims to answer the following main study question:

Are physicians who experience higher levels of SAIS more likely to encounter workplace aggression?

Based on the main study question, following questions were formed:

1. Is SAIS associated with non-physical and physical aggression?
2. Is SAIS associated with non-physical aggression perpetrated both by patients and relatives, and co-workers and superiors?

 

## Methods

### Participants and procedure

Data for this study were drawn from the cross-sectional Finnish Physicians’ Working Conditions and Health 2019 -study. Participants were sampled randomly from the register of the Finnish Medical Association, which covers almost all of the Finnish physician population. The participants who were asked to participate to current study (*n*= 8000, 37.8% of the Finnish physician population) were approached via email or letter if email was unavailable. A total of 3513 (response rate = 43.9%) physicians completed the questionnaire. The representativeness of the sample was assessed by comparing sample and population characteristics. The differences were so small that it was judged that inferences about the Finnish physician population can be made (see, for example[31],). Out of those who completed the questionnaire, only physicians who were actively practicing were included in the study (*n* = 2944) in order to capture the current state of SAIS and work aggression. The physicians in the sample practiced medicine either in the public sector (covering health centers and hospitals) or in the private sector, such as in private clinics. Public healthcare is available to all permanent residents in Finland regardless of their financial situation. To enable comparable analyses, those who had incomplete demographic information (age, sex or working sector) or missing study variables were removed, which resulted in a final analytic sample of 2786 physicians. In this final sample, 67.4% of the participants were female, with a mean age of 45.4 years (sd = 11.2), and the majority worked in the public sector (76.2%).

### Measures

The questionnaire items for the study variables are presented in the additional file 1.

#### Workplace aggression

Experiences of both non-physical and physical workplace aggression were measured.

Non-physical workplace aggression was assessed with the following question: “Non-physical violence is defined by ongoing, repeating bullying, oppression or offensive behavior. Do you experience or have you experienced non-physical violence or bullying in your work during the last 12 months?”. If a participant answered positively, the origin or perpetrator of aggression was also inquired and the participant could choose any number of the four provided options: coworkers, patients, patient’s relatives, and supervisors. From these answers, three dichotomous variables for non-physical aggression were formed: non-physical aggression from any source (1 = yes; 0 = no); non-physical aggression from co-workers or superiors (1 = yes; 0 = no); and non-physical aggression from patients or their relatives (1 = yes; 0 = no). There were physicians who reported that they had not experienced non-physical aggression, but still reported a specific source of non-physical aggression. These answers were re-coded as “has experienced non-physical aggression”. This measure has previously been associated with reduced job satisfaction [31].

Physical workplace aggression was assessed with the following question: “Have you been exposed or threatened with physical violence during the last 12 months?”. Participants could choose from three options: “No” (1), “I’ve only been threatened” (2), “I’ve also been exposed to violence” (3). A dichotomous variable for physical aggression was formed (1 = yes (options 2 and 3, at least threatened with violence); 0 = no). This measure has previously been used and associated with increased turnover intentions and reduced job satisfaction [31].

A variable for experiencing any type of aggression was formed based on variables of non-physical and physical aggression (1 = yes (has experienced either non-physical aggression, physical aggression, or both), 0 = no).

#### Stress attributed to information systems (SAIS)

SAIS was assessed with the following question: “How often (during the past half-year period) have you been troubled by, worried about, or stressed about” with respect to following items: i) “constantly changing information systems” and ii) “difficult, poorly functioning IT equipment/ software”. Participants were asked to estimate the level of frequency with a Likert scale from 1 (Very often or constantly) to 5 (Very rarely or never). SAIS was measured with the mean of the two items and the scale’s reliability was good in the present sample (Spearman-Brown reliability estimate = 0.76). This measure of SAIS has been previously used in a longitudinal study where it showed good reliability [19], and it has also previously been associated with lower work ability, higher levels of distress and lower self-estimated health [32].

#### Covariates

All analyses were adjusted for gender (1 = male, 2 = female), age, and work sector (1 = public, 2 = private), because demographic factors have been associated with a risk of aggression in some previous studies [2–6, 32].

### Statistical analyses

First, the bivariate associations were studied with Pearson correlation for SAIS and age, and with t-tests for SAIS other demographic variables (gender and work sector). Then, the differences in SAIS, age, gender, and work sector were analyzed with t-tests and $X^{2}$-tests in relation to experiencing a type of aggression (any type of aggression, physical, non-physical, non-physical by patients or relatives, non-physical by co-workers or superiors). Then logistic regression analysis was used to determine how different levels of SAIS were associated with the likelihood of experiencing different types of aggression. The independent variable was the mean score for SAIS. Dependent variables included any type of aggression, physical aggression, non-physical aggression, non-physical aggression perpetrated by patients or relatives, and non-physical aggression perpetrated by co-workers or superiors. Each of the aggression types was examined separately in their own models. The models were adjusted for demographic variables (gender, age, and work sector). The results for logistic regression were reported with Odds ratios (OR). The analyses were conducted using R version 3.6.3.

## Results

### Descriptive and bivariate associations

A fifth (23.4%, *n* = 651) of the physicians had experienced some type of workplace aggression within the last 12 months. Experiencing non-physical aggression (14.0%, *n* = 389) was somewhat more common than physical aggression (13.0%, *n* = 362), with 3.6% having experienced both. Experiencing non-physical aggression perpetrated by co-workers and superiors (9.8%, *n* = 272) was more common than non-physical aggression perpetrated by patients or relatives (5.0%, *n* = 139). A total of 1.1% (*n* = 32) physicians reported experiencing aggression both from patients and relatives and co-workers and superiors.

Higher SAIS was significantly associated with older age (*r* = 0.08, *p* < 0.001). Moreover, higher levels of SAIS were reported by females (mean = 3.40, difference = -0.12, t = -2.95, *p* = 0.003), and by those working in the public sector (mean = 3.43) compared to those working in the private sector (mean = 3.08, t = 7.61, *p* < 0.001).

The differences between those who reported experiencing some type of aggression and those who did not in SAIS and demographic variables are shown in the Table 1. Those who reported experiencing any type of aggression reported significantly higher levels of SAIS. Those who reported experiencing any type of aggression or physical aggression were younger. Instead, those that reported experiencing non-physical aggression or non-physical aggression from co-workers or superiors were older. Those that experienced aggression were more commonly females in all types of aggression except in physical aggression and non-physical aggression perpetrated by patients or relatives. Similarly, those that experienced aggression worked more commonly in the public work sector in all types of aggression except non-physical aggression perpetrated by patients and relatives.

**Table 1:** Descriptive statistics (*n* = 2786)

	Aggression^a^				Physical aggression^b^				Non-physical aggression^c^				Non-physical aggression by patients and relatives^d^				Non-physical aggression by co-workers and superiors^e^			
	No	Yes			No	Yes			No	Yes			No	Yes			No	Yes		
**Variable**	**Mean (sd)**		**t**	**p**	**Mean (sd)**		**t**	**p**	**Mean (sd)**		**t**	**p**	**Mean (sd)**		**t**	**p**	**Mean (sd)**		**t**	**p**
**SAIS**^**d**^	3.3 (1.04)	3.5 (1.05)	-4.33	**<.001**	3.33 (1.04)	3.48 (1.06)	-2.51	**.01**	3.31 (1.04)	3.57 (1.06)	-4.55	**<.001**	3.34 (1.04)	3.52 (1.07)	-1.95	.05	3.32 (45.1)	3.6 (1.06)	-4.11	**<.001**
**Age**	45.7 (11.3)	44.3 (11.1)	2.7	**.007**	45.9 (11.2)	41.8 (11.2)	6.5	**<.001**	45.2 (11.4)	46.8 (10.4)	-2.84	**.005**	45.4 (11.2)	44.2 (11.2)	1.26	.21	45.1 (11.4)	48.1 (9.81)	-4.71	**<.001**
**Variable**	**%**		**t**	**p**	**%**		**t**	**p**	**%**		**t**	**p**	**%**		**t**	**p**	**%**		**t**	**p**
**Gender**^**e**^** (women)**	65.7	73.3	12.8	**<.001**	67.2	68.8	0.27	.60	65.8	77.4	19.8	**<.001**	67.1	74.1	2.64	.10	66.1	80.1	21.52	**<.001**
**Work sector**^**f**^** (public)**	75.2	82.3	35.44	**<.001**	74.2	89.2	38.27	**<.001**	75.1	82.5	9.65	**.002**	75.9	80.6	1.32	.25	74.5	82.7	6.74	**.01**

^a^Any type of aggression, physical or non-physical 0 = no, 1 = yes

^b^Physical agression 0 = no, 1 = at least threatened

^c^Non-physical agression 0 = no, 1 = yes

^d^Stress attributed to information systems (1–5), 1 = low, 5 = high

^e^Gender 0 = male, 1 = female

^f^Work sector 1 = public, 2 = private

Bolded *p*-values indicate statistical significance

### The results of logistic regression

**Fig. 1:**
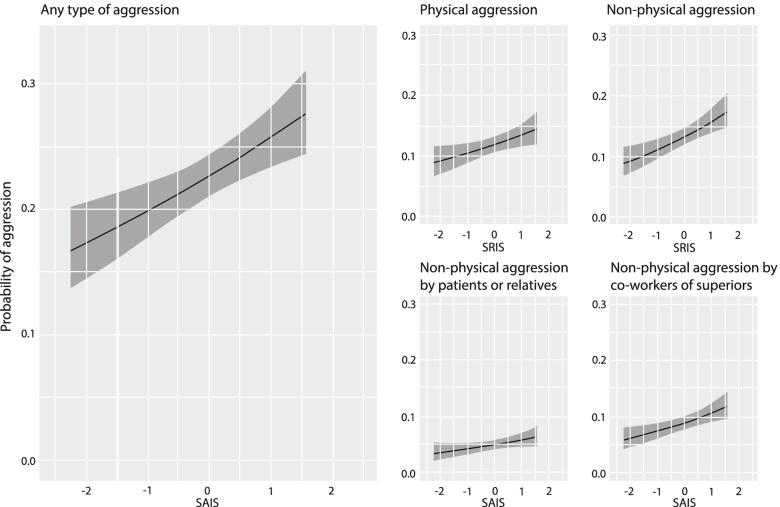
Predicted probabilities of experiencing aggression by levels of SAIS. The shaded area is for 95% confidence interval. SAIS scores were standardized for the figure. Predictions were derived from logistic regression analysis, adjusted for gender, age, and work sector

According to the results of logistic regression, SAIS was significantly associated with increased probability for encountering aggression with regard to all the aggression types except non-physical aggression perpetrated by patients or relatives (Table 2). SAIS had the strongest association with non-physical aggression, especially when aggression was perpetrated by co-workers or superiors. Overall, the results of unadjusted and adjusted models were quite similar. Model predicted probabilities for experiencing a specific type of aggression at different levels of SAIS are presented in Fig. 1.

**Table 2:** The results of logistic regression models

	**Aggression**		**Physical aggression**		**Non-physical aggression**		**Non-physical by patients or relatives**		**Non-physical by co-workers or superiors**	
Predictors	Odds ratio (95% CI)	p	Odds ratio (95% CI)	p	Odds ratio (95% CI)	p	Odds ratio (95% CI)	p	Odds ratio (95% CI)	p
Unadjusted										
SAIS	1.22 (1.11 – 1.33)	** < .001**	1.15 (1.03 – 1.29)	**.012**	1.29 (1.15 – 1.44)	** < .001**	1.19 (1.00 – 1.41)	0.05	1.31 (1.15 – 1.49)	** < .001**
Adjusted										
SAIS	1.18 (1.08 – 1.30)	** < .001**	1.15 (1.03 – 1.30)	**.015**	1.23 (1.10 – 1.37)	** < .001**	1.18 (0.99 – 1.42)	0.06	1.22 (1.07 – 1.40)	**.005**

Bolded *p*-values indicate statistical significance

Adjusted: adjusted for gender, age and work sector

*n*=2786, Statistical significance of regression coefficients was tested with Walds test

## Discussion

This cross-sectional study among Finnish physicians showed that higher levels of SAIS were associated with higher likelihood of being subjected to all types of aggression, except non-physical aggression perpetrated by patients or relatives. The association was most pronounced with non-physical aggression.

Our results build on the previous knowledge on the negative effects of SAIS in healthcare work environment and highlight the possible role of SAIS in increasing the risk of encountering aggression. Poorly functioning and constantly changing information systems have previously been shown to act as stressors for physicians [11, 14, 17, 18], and work stress has been linked with increased risk of workplace aggression [5–7]. However, to our knowledge, this is the first study to inspect the association between SAIS and experiencing workplace aggression. Potential mechanisms behind this association between SAIS and aggression could, for example, be that SAIS arouses frustration, negative affect, and aggressive inclinations in physicians; and the negative affect and inclinations could transfer to patients and staff through social contagion and learning. Previous theories of aggression [25–27] support these suggestions. However, since these mechanisms could not be examined in the current study, they should be examined in future studies, preferably in a longitudinal setting.

Because the current study examined cross-sectional data, it is also possible that experiencing aggression increases SAIS, and not the other way. As shown previously, experiencing aggression is associated with negative psychological outcomes (e.g. burnout, emotional exhaustion and depression) for the healthcare professional, and these outcomes have a negative impact on the work functioning and performance of healthcare professionals [1, 3]. Problems in work functioning and performance may lead to more mistakes or increased time pressure. In addition, emotionally exhausted physicians are less able to engage in positive teamwork [33]. In turn, teamwork, physician workload, and time pressure have been previously associated with higher levels of SAIS [12]. Thus, emotional and psychological stress caused by workplace aggression may leave physicians with fewer resources to cope with difficult and poorly functioning information systems and cause them to experience working with these systems as more stressful. Indeed, there is evidence that workplace aggression and psychological distress work in a vicious cycle, where they reinforce their negative effects through feedback loops [34, 35], a phenomena which has been observed between workplace aggression and work stress as well [7].

The association between SAIS and aggression appeared to be most pronounced with non-physical aggression. This finding is consistent with the results of a previous longitudinal study where stress-related variables were found to be better predictors of non-physical aggression than physical aggression [7]. The association of SAIS with non-physical aggression could be explained by the fact that the stressed behavior likely includes more aspects from non-physical aggression (e.g. angry tone of voice) than physical manifestations, since non-physical aggression is overall more common [2, 5]. Therefore, the modeled behavior [26] is also more likely to reflect aspects of non-physical aggression. Moreover, acts of physical aggression are perceived less morally acceptable than acts of non-physical aggression, which might make non-physical aggression a more likely response to SAIS overall. It should be noted that overall, the observed associations were weak, which reflects the fact that reasons underlying instances of aggression are complex and encompass numerous other factors that could not be examined in the current study.

No association was found for non-physical aggression perpetrated by patients or relatives, which might be due to the fact that relatively few physicians reported experiencing non-physical aggression by patients or relatives. Indeed, aggression by co-workers or superiors was found to be more prevalent than that by patients or their relatives. Previous studies have found the prevalence to be the other way around [6], although it is important to notice that studies concerning aggression perpetrated by superiors and co-workers is more scarce than that perpetrated by patients and relatives. The finding that aggression perpetrated by superiors and co-workers was more prevalent might reflect the influence of organizational climate on SAIS and aggression, because organizational climate has previously been connected with lower work stress [36] and overall psychological health[37]. Moreover, given that the same poorly functioning IS are used constantly in the same environment, SAIS might form a constant cycle of negative affect that feeds itself among the workforce. These results accentuate the need to investigate SAIS and workplace aggression more thoroughly on organizational or department level.

The demographic factors did not have a noticeable influence on the association between SAIS and aggression. However, work-sector, gender, and age were significantly associated with aggression. Experiencing aggression was more common among females and in public work sector, which is in line with previous literature (e.g [3].). However, findings related to age were here somewhat contradictory: experiencing some types of aggression was more common among younger physicians and others among older physicians. Previous findings on aggression and age have similarly been contradictory [2, 6, 38].

This study is subject to some limitations, which should be considered when interpretating the results. First, it should be noted that causational direction between SAIS and aggression could not be determined as the current study used cross-sectional data. Secondly, there were limitations related to the study variables. SAIS was measured as a mean of only two items that asked about negative reactions to information systems. It should be noted that our measurement of stress attributed to information systems (defined as “troubled by, worried about or stressed about”) more accurately depicts negative psychological reactions or psychological distress attributed to information systems. Thus, our definition of stress follows the model set by Posner and colleagues [39] to define stress as a negatively valued emotional state that is characterized by high arousal. As such, it differs from definitions used in work stress literature that use work stress to refer to the process of work-related stimuli leading not only to negative psychological but behavioral and physical consequences as well [40]. In addition, all the variables were assessed only on an individual level and no information was collected of participants’ department or organizational belonging. This means that we measured subjective, individual-level perceptions of information systems and workplace aggression, and could not examine, for example, whether physicians working in a same department or organization had similar perceptions. In addition, using self-report measures might have introduced common-methods variance in the results. Since workplace aggression was measured with subjective reports rather than objective measures, it is also possible that stress due to high workload might influence a physician to perceive the behavior of others as aggressive (mood-congruity) [41]. Similarly, high workload might increase SAIS by making it impossible to participate in IS user training, which could result in low IS competence and frustration. It is also possible that high workload induces stress reactions that lower the tolerance towards poorly functioning IS. Future studies with more objective measurements of workplace aggression, workload and IS quality could help shed light on these associations and help disentangle questions related to cause-effect relationships. Lastly, the current study was conducted with physicians, so the results should be applied to other healthcare professionals with caution.

## Conclusion

To conclude, the current study provides evidence that higher levels of SAIS are associated with higher likelihood of experiencing aggression at workplace. Because SAIS may increase the risk of experiencing aggression, it is possible that SAIS also endangers the wellbeing of physicians and thereby the quality of patient care. Poorly functioning information systems are, of course, not the only frustrating factors in the healthcare work environment. It has also been shown that, for example, unfair managerial procedures, high job strain and job demands can provoke aggressive behavior [42, 43]. In addition, the effect sizes of our bivariate differences and Odds ratios are small, thus our results should be interpreted with caution. However, even small effects should be considered, since it has been suggested that even though the size of the effect in psychological research would be very small, it may potentially be very consequential in the long run [44]. The present results align with previous literature on the potential negative effects of SAIS. This notion has decisive implications for the future development of information systems, especially since over a third of physicians in our study reported suffering from SAIS frequently or more often, in line with previous literature [14, 17, 18]. Improvements to both the wellbeing of physicians and the quality of patient care could be gained in the future by including proper usability design and active participation of physicians in the development of IS. Healthcare organizations should offer easy-to-use systems without technical problems, as that would promote the workflow and release time for patient work. Moreover, resourcing additional time and training for physicians during introduction of a new IS could also alleviate time pressure and thus stress attributed to managing new IS. Special care should be taken in ensuring that IS functions more as a resource than a demand for the healthcare workers in the future. Similar measures should be heavily considered outside of Finland as well, since IS have been consistently reported to cause stress for physicians in other countries as well (e.g [17, 18].) along with high prevalence rates of workplace aggression in healthcare (e.g [3].). However, the current findings warrant more research, especially regarding the possible mediating factors between SAIS and aggression. The role of organizational climate and general workload in arousing stress and consequent SAIS and aggression should be examined as well. Collecting longitudinal information on the effect of SAIS on different types of aggression on organizational or department level with different professional groups could provide further insights in future research.

## Supplementary Information

**Additional file 1. **Measures used in the study.

## Data Availability

The data that support the findings of this study are available from Finnish Medical Association, but restrictions apply to the availability of these data, which were used under license for the current study, and so are not publicly available. The pseudonymized questionnaire data can however be shared by request from the authors after approval of the study group and Finnish Medical Association.
