# Supplementary material for: The association between stress attributed to information systems and the experience of workplace aggression: a cross-sectional survey study among Finnish physicians
Source: BMC Health Serv Res. 2022 May 31;22:724. doi: 10.1186/s12913-022-08116-w (PMC9158205; doi:10.1186/s12913-022-08116-w)
Supplement: Supplementary file 1 — Additional file 1. Measures used in the study. [file 12913_2022_8116_MOESM1_ESM.docx]

**Additional file 1**

**Measures used in the study**

**Independent variable**

***Stress attributed to information systems (SAIS):***

*How often have you been troubled by, worried about, or stressed about the issues mentioned below during the past 6 months?*

1. Constantly changing information systems

2. Difficult, poorly functioning IT equipment/software

Response options:

1. Never

2. Very rarely

3. Quite rarely

4. Quite often

5. Very often

6. Constantly

**Dependent variables**

***Non-physical aggression:***

*Non-physical violence is defined by ongoing, repeating bullying, oppression, or offensive behavior. Do you experience or have you experienced non-physical violence or bullying in your work during the last 12 months?*

Response options:

1. No

2. Yes

*If you have experiences or you currently experience non-physical aggression at your work, by whom?*

Response options:

1. Co-workers

2. Patients

3. Patient’s relatives

4. Superior

***Physical aggression:***

*Have you been exposed or threatened with physical violence during the last 12 months?*

Response options:

1. No

2. I have only been threatened

3. I have also been exposed to violence
